# Clinical analysis of 8 cases of intramural pregnancy after embryo transfer

**DOI:** 10.1097/MD.0000000000048690

**Published:** 2026-05-15

**Authors:** Juan Wang, Fengxia Liu, Yourong Cao, Bin Zhang, Xiaohong Li, Arshad Mehmood, Yi Mo

**Affiliations:** a Gynecology Department, The Reproductive Hospital of Guangxi Zhuang Autonomous Region, Nanning, China; b Reproductive Department, The Reproductive Hospital of Guangxi Zhuang Autonomous Region, Nanning, China; c The Reproductive Hospital of Guangxi Zhuang Autonomous Region, Nanning, China; d State Key Laboratory of Digital Medical Engineering, School of Biological Science and Medical Engineering, Southeast University, Nanjing, China.

**Keywords:** clinical features, embryo transfer, infertility, intramural pregnancy, treatment

## Abstract

This study aimed to improve the diagnosis and treatment of intramural pregnancy (IMP). A retrospective analysis was conducted of 8 cases of IMP admitted between November 2016 and June 2024. Data on patients’ clinical characteristics, diagnosis, treatment, and prognosis were systematically reviewed. All 8 patients experienced IMP following embryo transfer due to tubal factor infertility. Among them, 2 had adenomyosis. All patients had a history of pelvic surgery, including 4 with tubal pregnancy surgery and 2 with uterine cavity surgery. A total of 11 embryos were transferred among the 8 patients, with 8 being high-quality embryos. The fertilization methods included in vitro fertilization and intracytoplasmic sperm injection. The onset of symptoms occurred between 23 and 54 days after embryo transfer. Upon admission, none of the 8 patients exhibited obvious abdominal pain. Preoperative diagnosis included 5 cases of tubal pregnancy and 3 cases of IMP. There were no cases of uterine rupture prior to treatment. All cases were confirmed via combined hysteroscopic and laparoscopic exploration. Among the cases, 6 intramural pregnancy lesions protruded toward the uterine serosa, 1 slightly into the uterine cavity, and 1 showed no protrusion into either the uterine cavity or the serosa. Cases with gestational lesions slightly protruding into the uterine cavity primarily underwent hysteroscopic resection of the gestational lesion, whereas lesions protruding into the uterine serosa were treated with laparoscopic resection. For cases where the lesion neither protruded into the uterine cavity nor into the serosa, ultrasound-guided local methotrexate (MTX) injection was the primary treatment approach. These cases either received or did not receive systemic MTX treatment as adjuvant therapy. After treatment for gestational lesions, no additional surgeries were performed, and serum human chorionic gonadotropin levels returned to normal within 20 to 90 days. IMP is a rare condition associated with a risk of uterine rupture. Clinical presentations are often atypical, and imaging studies are critical for early diagnosis. Treatment approaches are individualized and may involve surgical resection of gestational lesions, ultrasound-guided embryonic reduction, or local/systemic administration of MTX.

## Graphical abstract

**Figure:**
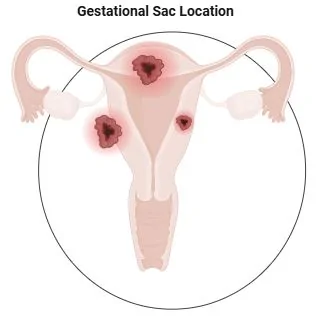

**Key points**

- All 8 cases of IMP occurred after embryo transfer, with atypical clinical presentations and delayed symptom onset.
- All patients had a history of pelvic or uterine surgeries, emphasizing uterine trauma as a key risk factor.
- Lesions varied in their protrusion into the uterine cavity or serosa, guiding the choice of hysteroscopic, laparoscopic, or medical treatment.
- Treatment included surgical resection, ultrasound-guided MTX injection, and systemic therapy, with individualized approaches based on lesion type.
- Five patients achieved full-term pregnancies posttreatment, highlighting the success of conservative and tailored management in IMP.

**Significance statement**

IMP is a rare and potentially life-threatening form of ectopic pregnancy (EP) that poses diagnostic and therapeutic challenges following assisted reproductive technology (ART). This study provides one of the largest clinical analyses of embryo transfer-related IMP, underscoring the importance of early imaging-based diagnosis, the role of prior uterine interventions, and the effectiveness of individualized management. These findings contribute critical insights for optimizing outcomes and fertility preservation in high-risk patients.

## 1. Introduction

Intramural pregnancy (IMP) is a rare form of ectopic pregnancy (EP), referring to the implantation of a gestational sac within the uterine muscle wall without communication with the uterine cavity or tubes, surrounded by myometrial tissue.^[1]^ Its incidence accounts for ≤1% of all EPs.^[2]^ This trend underscores the critical need for heightened awareness and improved diagnostic strategies for atypical implantation sites.^[3]^ Key risk factors include previous uterine trauma from procedures, such as curettage, myomectomy, cesarean section, or hysteroscopic surgery, which create microscopic tracts or defects facilitating abnormal implantation.^[4]^ Adenomyosis has been implicated, as ectopic endometrial tissue within the myometrium undergoes decidualization, providing a nidus for embryo implantation.^[5]^ In the context of assisted reproductive technology (ART), technical factors during embryo transfer, such as catheter placement and uterine contractility, may contribute to IMP.^[6]^ IMP poses diagnostic and therapeutic challenges. Its early presentation is asymptomatic or characterized by mild, nonspecific symptoms, such as light vaginal bleeding, leading to misdiagnosis as a tubal pregnancy or a failing intrauterine gestation.^[7]^ This diagnostic delay carries the grave risk of catastrophic uterine rupture, resulting in life-threatening hemorrhage, emergency hysterectomy, and loss of future fertility.^[8]^ Therefore, early and accurate diagnosis, predominantly reliant on high-resolution transvaginal ultrasound and occasionally magnetic resonance imaging (MRI), is paramount.^[9]^ Current management strategies for IMP are diverse and individualized, reflecting the lesion’s size, location, serum beta human chorionic gonadotropin (β-HCG) levels, and the patient’s desire for fertility preservation. Options range from surgical interventions, including hysteroscopic, laparoscopic, or laparotomic resection, to medical management with systemic or locally injected methotrexate (MTX), and minimally invasive approaches like ultrasound-guided fetal reduction or uterine artery embolization.^[10]^ However, due to the extreme rarity of IMP, evidence is largely confined to case reports and small series, and there is no consensus on an optimal treatment algorithm for cases arising after embryo transfer.

This study investigates the clinical characteristics of 8 cases of IMP following embryo transfer to explore clinically appropriate management strategies and reduce misdiagnosis rates.

## 2. Materials and methods

### 2.1. Participants

The clinical data of 8 cases of IMP diagnosed via transvaginal ultrasound, combined with hysteroscopy and laparoscopy in patients who were admitted to The Reproductive Hospital of Guangxi Zhuang Autonomous Region from November 2016 to June 2024, were retrospectively analyzed.

### 2.2. Methods

A retrospective collection of clinical data included age, body mass index (BMI), and menstrual cycle. The criteria for determining regular menstruation were a menstrual duration of <7 days and a menstrual cycle length between 21 and 35 days.^[11]^ Dysmenorrhea severity was assessed using the visual analog scale pain score, with a total score ranging from 0 to 10 and categorized into 3 levels: mild pain (≤3 points), tolerable; moderate pain (4–6 points), affects sleep but is still tolerable; and severe pain (7–10 points), significantly impacts sleep and appetite, difficult to tolerate.^[12]^ BMI (kg/m^2^) = weight/height^2^.^[13]^ Underweight: BMI < 18.5 kg/m^2^; normal weight: 18.5 ≤ BMI < 24 kg/m^2^; overweight: 24 ≤ BMI < 28 kg/m^2^; and obese: BMI ≥ 28 kg/m^2^.^[14]^

#### 2.2.1. Type and duration of infertility

The types of infertility in patients were primary infertility and secondary infertility. Primary infertility: the patients were women who had no history of pregnancy and had sexual intercourse without contraception for at least 12 months without becoming pregnant. Secondary infertility: the patients were women who had experienced a previous pregnancy and then had sexual intercourse without contraception for at least 12 months without becoming pregnant.^[15]^

#### 2.2.2. Indications for ART

- *Pre-ART ovarian reserve function assessment (baseline hormones + antral follicle count*)
- *ART cycle details (transfer cycles, embryo fertilization method, embryo characteristics*)

The embryo transfer cycle refers to the process of preparing the endometrium and transferring embryos into it. This can be categorized as follows:
  1. *Fresh embryo transfer after controlled ovarian hyperstimulation (COH*): transfer of embryos created during the same cycle of ovarian stimulation.
  2. *Frozen-thawed embryo transfer (FET*): embryos are thawed and transferred in a subsequent cycle.
  3. Endometrial preparation during the thawing cycle includes:
    *Natural cycle*: utilizing the patient’s spontaneous menstrual cycle.
    *Hormone replacement cycle (HRT*): synthetic hormones (estrogen, progesterone) are used to prepare the endometrium.
    *Ovulation induction cycle*: medications to stimulate ovarian activity and endometrial growth.
    Endometrial thickness was the measurement taken before transplantation (human chorionic gonadotropin [HCG] day/endometrial thickness), which is translated contextually.
    The main embryo fertilization methods are in vitro fertilization (IVF) and intracytoplasmic sperm injection (ICSI).
    Embryo status and grading criteria^[16]^: Day-3 (D3) embryos: cleavage-stage embryos (D3) evaluated using the Istanbul Consensus scoring system. High-quality cleavage-stage embryos are defined as those graded level 1 or 2 on D3 with ≥7 blastomeres (cells). D5/D6 embryos: blastocyst-stage embryos, assessed via the Gardner scoring system, which evaluates degree of blastocyst expansion and hatching, size/compactness of the inner cell mass (ICM), and compactness/cellularity of the trophectoderm (TE). High-quality blastocysts are classified as ≥3BB grade: both ICM and TE scores must be B or higher (3BB or above). Low-quality blastocysts are graded 3 to 6 (AC/BC/CA/CB): indicate inferior ICM/TE quality based on scoring combinations (AC = ICM A/TE C; BC = ICM B/TE C).^[17,18]^

- *Embryo transfer procedure (embryo transfer tubes, process*): according to the presence or absence of mucosal blood staining in the catheter during embryo transfer.
- *Luteal phase support regimen post-transfer*: after embryo transfer, patients continue to use luteal support medications until weeks 10 to 12 of pregnancy.^[19]^
- *Clinical manifestations, imaging features, intraoperative exploration findings, and treatment modalities*

#### 2.2.3. Diagnosis and treatment

*Diagnostic criteria*: elevated blood HCG after embryo transfer, absence of a gestational sac in the uterine cavity on ultrasound, or surgical confirmation that the gestational sac was located within the myometrium of the uterus.

Treatment included surgical resection, ultrasound-guided MTX injection, and systemic therapy.

*Cure criteria*: blood HCG levels return to normal, the intramural pregnancy lesion disappears, and there are no clinical symptoms such as abdominal pain.

## 3. Results

### 3.1. General data

All 8 cases were patients who underwent assisted reproductive embryo transfer for pregnancy because of infertility at The Reproductive Hospital of Guangxi Zhuang Autonomous Region from November 2016 to June 2024. Their ages ranged from 28 to 40 years, with a median age of 35 years. Among them, 5 had primary infertility, and 3 had secondary infertility. The duration of infertility ranged from 1 to 9 years, with a median of 4 years. BMI values ranged from 16.9 to 29.4, with a median of 25.5. Four cases were classified as overweight, and 1 as obese.

Among the 8 patients, 1 had polycystic ovary syndrome with oligomenorrhea, whereas the remaining 7 had regular menstrual cycles. The primary indication for ART in all 8 patients was infertility due to tubal factors. Pre-ART assessments showed normal ovarian reserve function in all cases. Regarding dysmenorrhea severity: 3 patients had severe dysmenorrhea, 3 had mild dysmenorrhea, and 2 had no dysmenorrhea. Two of the 8 patients had adenomyosis. All 8 patients had a history of prior surgeries, including laparoscopic salpingostomy and salpingoplasty, EP surgery, appendectomy, cesarean delivery, hysteroscopy, endometrial polypectomy, and hysteroscopic adhesiolysis for intrauterine adhesions. There were no cases with a history of repeated curettage. In the present cohort (Table 1), all 8 patients had undergone previous pelvic or uterine interventions, supporting the association between uterine trauma and the development of IMP.

**Table 1:** General clinical characteristics of 8 patients diagnosed with IMP.

No.	Age	Dysmenorrhea severity	Gravida/para (G/P)	Infertility type	Infertility period (yr)	Surgical history	BMI	Year of IMP onset
1	34	Mild	G0P0	Primary infertility	8	Laparoscopy (2015: tubal adhesion correction)	27.2	2016
2	35	Moderate-severe	G3P1	Secondary infertility	1	3 surgeries for ectopic pregnancy; bilateral salpingectomy (2017)	24.3	2020
3	35	Secondary, severe	G0P0	Primary infertility	2	Laparoscopy + hysteroscopy (2019: tubal reconstruction, hysteroscopy)	23.2	2021
4	29	Mild	G0P0	Primary infertility	5	Laparoscopy for hydrosalpinx (2019), left salpingectomy (2021)	16.9	2021
5	28	None	G0P0	Primary infertility	7	Hysteroscopy for Asherman syndrome (2021)	27.7	2021
6	39	Severe	G3P1	Secondary infertility	3	Cesarean section (2016), salpingectomies for ectopic pregnancies (2017–2018), and bilateral tubal ligation	26.7	2022
7	35	None	G3P1	Secondary infertility	1	Cesarean section (2007), laparoscopy for ectopic pregnancy (2013), appendectomy (2022)	29.4	2022
8	40	Mild	G0P0	Primary infertility	2	Hysteroscopy for endometrial polypectomy (November 2023)	23.78	2024

IMP = intramural pregnancy.

### 3.2. Embryo transfer details

*Patient details*: Eight patients underwent embryo transfer procedures performed by different qualified senior physicians with senior professional titles. The embryo transfer catheters used were either the Prodimed catheter (France) or the Cook catheter (United States). No blood staining was observed on the catheters post-procedure.

*Endometrial thickness*: Measured on the day of HCG administration or endometrial conversion day, uterine endometrial thickness ranged from 8 to 12 mm, with a median of 8.7 mm.

*Embryo transfer cycles*: Included COH cycles (2 cases), HRT (2 cases), down-regulated HRT (2 cases) after leuprolide 3.75 mg, natural cycles (1 case), and ovulation induction cycles (1 case).

*Embryo transfer outcomes*: A total of 11 embryos were transferred among the 8 patients, including 8 high-quality embryos.

*Fertilization methods*: IVF and ICSI were used.

*Transfer specifics*: Two patients received D3 double fresh embryo transfers during the COH cycle. One patient with embryos generated via ICSI underwent a frozen double embryo transfer. The remaining 5 patients received a single FET. Embryo transfer protocols, fertilization modes, and embryo quality scores of the 8 patients are summarized (Table 2). Most cases underwent IVF with high-quality D3 or D5 embryos, and all transfers were performed using standard catheters under optimized endometrial conditions.

**Table 2:** Embryo transfer characteristics of 8 cases diagnosed with IMP.

No.	Fertilization mode	Embryo number	Transfer cycle	Embryo type	Embryonic period	Embryo score	Transfer tube	Endometrium thickness (mm)
1	IVF	2	Oocyte retrieval cycle (COH)	Fresh	D3	6CG1, 6CG2	Prodimed	12
2	IVF	2	Oocyte retrieval cycle (COH)	Fresh	D3	7CG1, 8CG2	Prodimed	10.8
3	IVF	1	Down-regulated HRT	Frozen	D5	5BC	COOK (with liner)	8
4	IVF	1	HRT	Frozen	D5	5BB	COOK	9.1
5	IVF	1	HRT	Frozen	D5	5AB	COOK	8
6	IVF	1	Down-regulated HRT	Frozen	D5/D6	5BB	COOK	9.1
7	ICSI	2	Ovulation induction cycle	Frozen	D5	4BC	COOK	8.1
8	IVF	1	Natural cycle	Frozen	D5	5AB	COOK	8.2

COH = controlled ovarian hyperstimulation, down-regulated HRT = HRT after Leuprolide 3.75 mg, cleavage-stage embryo–blastocyst, Fresh embryo = fresh embryo transfer, frozen embryo = frozen embryo transfer, endometrium thickness is the endometrial thickness before transplantation (HCG administration or endometrial conversion day), HCG = human chorionic gonadotropin, HRT = hormone replacement cycle, ICSI = intracytoplasmic sperm injection, IVF = in vitro fertilization.

### 3.3. Clinical characteristics

*Symptoms at admission*: All 8 patients presented without significant abdominal pain. Two patients (Cases 1 and 5) had vaginal spotting, occurring 54 days and 25 days post-embryo transfer, respectively. No uterine ruptures were reported prior to treatment.

#### 3.3.1. Diagnosis

*Primary diagnostic method*: Ultrasound. Five patients were diagnosed with tubal pregnancy, 3 patients were diagnosed with IMP. Three cases showed a fetal heartbeat on ultrasound.

*Diagnosis timing*: IMP was diagnosed between 23 and 54 days post-embryo transfer, with a median of 27.5 days. The clinical symptoms, imaging features, and preoperative diagnoses of the 8 patients with intramural or interstitial pregnancy are summarized (Table 3). Most cases were identified incidentally during routine ultrasound evaluation following embryo transfer, with atypical presentations, such as absence of abdominal pain but presence of adnexal or intramural masses.

**Table 3:** Clinical characteristics of 8 cases diagnosed with IMP.

No.	Clinical symptom	Day post-embryo transfer	Admission ultrasound	Preoperative diagnosis
1	Vaginal bleeding for 2 d	54	Endometrial thickness: 3.5 mm. Right adnexal region: A mixed-type mass measuring 52 × 50 × 40 mm. Suspected to be an ectopic pregnancy (cornual tubal pregnancy).	Ectopic pregnancy
2	A pelvic adnexal mass was detected by B-mode ultrasound within half a day	25	Endometrial decidua thickness: 18.6 mm. Myometrium (left side): a gestational sac of 13 × 13 × 11 mm with a viable embryo, not communicating with the uterine cavity. Suspected to be an interstitial pregnancy of the left tube.	Interstitial pregnancy of the left tube
3	An ultrasound examination revealed adnexal masses half a day ago	28	Adenomyosis, endometrial thickness: 10.5 mm, gestational sac in the right cornual myometrium (25 × 24 × 20 mm) with embryo viability, including yolk sac and fetal cardiac activity. Suspected to be an interstitial pregnancy of the right tube.	Interstitial pregnancy of the right tube
4	An ultrasound examination revealed adnexal masses half a day ago	27	Endometrial thickness: 11.1 mm. Mixed echogenic mass in the left myometrium (31 × 29 × 29 mm), well-defined borders, protruding outward. Gestational sac located in the left myometrium (17 × 15 × 12 mm), with fetal heart activity. The mass is adjacent to the left cornual decidual tissue, with a distance of 1.4 mm from the serosal layer. No communication with the uterine cavity. Suspected to be an interstitial pregnancy of the left tube.	Interstitial pregnancy of the left tube
5	Vaginal bleeding for 4 d	25	Multiple uterine fibroids (largest dimensions: 15 × 14 × 13 mm and 15 × 12 × 9 mm). Endometrial decidual thickness: 13.6 mm, multiple small hypoechoic areas. A mixed echogenic mass in the fundal myometrium (measuring 18 × 15 × 14 mm), with one-third protruding into the uterine cavity. Cannot exclude intramural pregnancy. Left-sided hydrosalpinx.	Intramural pregnancy
6	An ultrasound examination revealed adnexal masses half a day ago	32	Adenomyosis, cesarean scar uterus, multiple uterine fibroids (largest: 17 × 16 × 14 mm), endometrial decidual thickness of 8.5 mm, right ovarian endometrioma (chocolate cyst: 15 × 14 × 14 mm), and a left adnexal mixed-type mass (12 × 12 × 11 mm), suspected ectopic pregnancy.	Interstitial pregnancy? Intramural pregnancy?
7	An ultrasound examination revealed adnexal masses half a day ago	23	Endometrial thickness: 12.8 mm. A slightly hyperechoic mass (14 × 12 × 10 mm) and a mixed echogenic mass within the right myometrium of the uterus, slightly protruding outward (suspected ectopic pregnancy).	Ectopic pregnancy
8	An ultrasound examination revealed adnexal masses half a day ago	44	Endometrial thickness of 17 mm. A mixed echogenic mass (23 × 22 × 18 mm) in the left uterine cornual myometrium is considered likely to represent an intramural pregnancy. Bilateral hydrosalpinx.	Intramural pregnancy

Days post-embryo transfer = days between embryo transfer and diagnosis of intramural pregnancy, IMP = intramural pregnancy.

### 3.4. Pregnancy location and treatment

**Figure 1.**
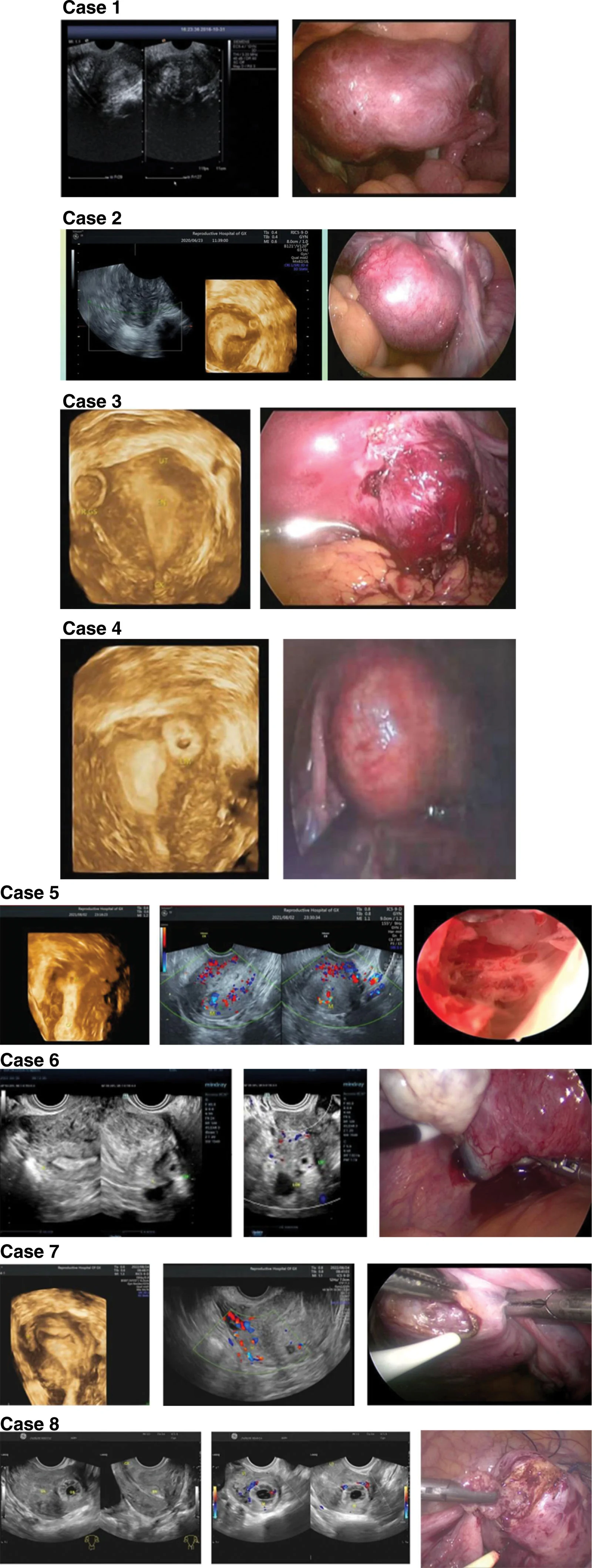
Preoperative ultrasound images and intraoperative surgical images (Cases 1–8).

Upon surgical exploration, among 8 patients, 1 case was located on the left wall of the uterus without protrusion toward either the serosa or the uterine cavity. Six cases involved the posterior myometrium protruding toward the serosa near the left/right cornua of the uterus. One case protruded toward the uterine cavity. Lesions were removed via laparoscopy or hysteroscopy. For Case 1: Initial laparoscopic exploration revealed a large anterior wall lesion (5 × 6 cm) with rich vascular supply. Diagnosed via laparoscopy, followed by local injection of 50 mg MTX. Systemic MTX administration occurred 1 week and 1 month post-surgery. At day 50 posttreatment (HCG: 106.85 mIU/mL), lower abdominal pain and lesion enlargement necessitated reoperation, with intraoperative bleeding of 450 mL. Other patients had intraoperative bleeding <100 mL. Case 2 underwent surgical exploration without identification of gestational lesions, followed by ultrasound-guided fetal reduction and sequential local or systemic MTX therapy. Not all patients required secondary surgery after lesion removal. Among 7 cases where gestational tissue from uterine muscle wall resection was sent for biopsy, 6 cases showed embryonic villous tissue. In 1 case, following hysteroscopic resection, there was extensive neutrophil infiltration without evidence of villi or trophoblastic cells. All 8 cases had negative findings for villous tissue on hysteroscopy and endometrial biopsy. Initial blood HCG levels at admission ranged from 1271 to 106,910 mIU/mL. Case 2 developed mild transaminase elevation, which normalized after liver-protective therapy. No significant hepatic, renal abnormalities, or blood count abnormalities were observed in other patients. Hospital stay duration: 20 to 70 days, with a median of 40.5 days. Three early cases had prolonged stays: 70, 90, and 67 days, respectively. HCG levels at presentation, lesion location, treatment modalities, and time to HCG normalization for the 8 IMP cases are summarized (Table 4). All patients received individualized treatment based on lesion characteristics, ranging from hysteroscopic or laparoscopic excision to local and systemic MTX administration. Ultrasound and surgical images of the 8 cases are presented (Fig. 1, Cases 1–8).

**Table 4:** Serum HCG levels, gestational site characteristics, and treatment strategies of 8 cases with IMP.

No.	HCG level at presentation (mIU/mL)	Gestational site	Treatment approach	Intraoperative bleeding (mL)*	HCG level at discharge (mIU/mL)	Hospital stay	Time to HCG normalization (d)†
1	61,638	Lower segment of the anterior right uterine wall protrudes toward the serosa	Combined hysteroscopic and laparoscopic examination + local MTX (50 mg) injection under direct laparoscopic visualization + systemic MTX (50 mg) administration twice + laparoscopic excision of the lesion + single systemic MTX (50 mg) administration	450	161.64	53	70
2	7274	The myometrium of the left uterine wall does not protrude into the endometrium or serosa	Combined hysteroscopic and laparoscopic exploration + ultrasound-guided fetal reduction (local injection of MTX 10 mg) + systemic MTX 60 mg	50	394.53	45	90
3	106,910	The right uterine myometrium near the cornua protrudes toward the serosa	Combined hysteroscopic and laparoscopic examination + laparoscopic excision of the lesion	100	2659	6	67
4	51,707	Left uterine myometrium near the cornua protrudes toward the serosa	Combined hysteroscopic and laparoscopic examination + laparoscopic excision of the lesion + local MTX (25 mg) injection under direct laparoscopic visualization	50	1652	8	38
5	9871	Posterior uterine myometrium slightly protrudes toward the endometrial cavity	Hysteroscopic excision of gestational lesions + systemic MTX 50 mg	10	343.6	5	30
6	1271	Left uterine myometrium near the cornua protrudes toward the serosa	Combined hysteroscopic and laparoscopic examination + laparoscopic excision of the lesion + local MTX (20 mg) injection under direct laparoscopic visualization	10	68.64	5	43
7	6855	The right uterine myometrium near the cornua protrudes toward the serosa	Combined hysteroscopic and laparoscopic examination + laparoscopic excision of the lesion + local MTX (50 mg) injection under direct laparoscopic visualization	20	450.84	13	20
8	10,734	Left uterine myometrium near the cornua protrudes toward the serosa	Combined hysteroscopic and laparoscopic examination + laparoscopic excision of the lesion + local MTX (50 mg) injection under direct laparoscopic visualization	10	2534	4	26

HCG = human chorionic gonadotropin, IMP = intramural pregnancy, MTX = methotrexate.

* Intraoperative bleeding = blood loss during the laparoscopic surgery performed during this hospitalization.

† Time to HCG normalization (day) = the number of days from the initiation of inpatient treatment until serum HCG levels returned to the normal range.

### 3.5. Follow-up results translation

*Decline of blood HCG levels*: Posttreatment, blood β-HCG levels returned to normal in all 8 patients within 20 to 90 days, with the longest duration observed in Case 2 (no surgical removal of gestational lesions).

*Postoperative ultrasound findings*: Two cases showed heterogeneous echogenicity at the uterine lesion sites. Six cases had no abnormal echogenicity in the uterine myometrium on follow-up scans.

*Subsequent pregnancies and outcomes*: *Cases 2, 4, 5, and 6*: achieved pregnancy via FET 12, 13, 7, and 16 months post-surgery, respectively.

*Case 2*: full-term vaginal delivery.
*Cases 4, 5, 6*: full-term cesarean deliveries.
*Case 1*: diagnosed with polycystic ovary syndrome.

*First FET attempt 1 year post-surgery*: failed to conceive. No further follow-up visits have been made to the hospital since then.

*Case 3*: diagnosed with adenomyosis. Spontaneous pregnancy 1 year post-surgery, but terminated due to right-sided tubal EP. No subsequent pregnancies reported.
*Case 6*: diagnosed with adenomyosis. No pregnancies reported despite 2 + years of follow-up post-surgery.
*Case 8*: underwent FET 6 months post-surgery, resulting in early missed abortion (nonviable pregnancy).

## 4. Discussion

IMP is a rare type of EP where the embryo implants within the uterine myometrium, isolated from the uterine cavity, fallopian tubes, or pelvic-abdominal cavities. Its incidence constitutes ≤1% of all EPs.^[20]^ If left undiagnosed early, advancing gestation can lead to uterine rupture, life-threatening hemorrhage, potentially necessitating hysterectomy or maternal mortality. Clinical reports indicate that some cases present with uterine rupture when the gestational mass reaches 4 to 5 cm or larger, causing an acute abdomen or hemorrhagic shock requiring emergency surgery.^[5,7,21]^ Currently, approximately 70 cases have been documented in the literature,^[22]^ emphasizing the critical need for early diagnosis and timely intervention as central to clinical management.

### 4.1. Pathogenesis

Some studies suggest that the development of IMP may be related to the following factors^[2]^: damage to the endometrium or myometrium, such as from hysteroscopic surgery, curettage, endometritis, or a history of cesarean section and myomectomy. These conditions disrupt the continuity and integrity of the endometrium or myometrium, enabling the embryo to implant into and develop within the uterine muscle layer, or during ART embryo transfer that penetrates the endometrial layer. Adenomyosis, in which adenomyosis or adenomyoma can form sinus tracts through which the embryo implants into the myometrium, is also implicated. The ectopic endometrium undergoes decidualization under the influence of estrogen and progesterone, facilitating further embryonic development.

With the application of ART, the incidence of EP has increased. The incidence of EP after IVF-ET is statistically reported to range from 2.1% to 9.4%, accounting for 2% to 11% of all pregnancies following embryo transfer.^[23]^ Among these, the incidence of tubal ectopic pregnancies is 2% to 5%.^[24]^ A history of EP is a high-risk factor for recurrent EP, with an approximate recurrence rate of 10%.^[25]^ Some studies suggest that a history of tubal ectopic pregnancies does not increase the risk of subsequent EP following embryo transfer.^[26]^ Currently, there is no literature reporting whether embryo transfer increases the risk of IMP.

In the present study’s 8 cases, 5 were primary infertility, and 3 were secondary infertility. Among them, 6 were complicated by moderate to severe dysmenorrhea, while the 2 patients without dysmenorrhea were diagnosed with secondary infertility and primary infertility, respectively. Adenomyosis was present in 2 cases, and all 8 cases had a history of prior surgical procedures. These included isolated combined laparoscopy and hysteroscopy (tubal fimbriectomy), cesarean section, history of tubal pregnancy surgery, or appendectomy. Among these, 4 cases had a history of tubal pregnancy surgery, including 2 cases of recurrent tubal pregnancies, for which patients had undergone either salpingectomy or tubal ligation.

As of now, there are no existing reports on the correlation between IMP and the endometrial status at the time of embryo transfer, the number of embryos transferred, embryonic quality, or embryonic stage.

In the present study’s cases, the embryos involved in IMP following embryo transfer were fertilized via IVF (9 embryos) and ICSI (2 embryos). Among 11 embryos, 9 were high-quality embryos. Two cases of cleavage embryos were transferred in fresh embryo transfer cycles. The remaining cases involved blastocyst transfer in FET cycles.

The embryo transfer cycles in the present study included the COH cycles (ovum retrieval cycles), HRT, down-regulated HRT, natural cycles, and ovulation induction cycles.

All embryo transfer procedures were performed by different experienced physicians. Notably, 1 patient with adenomyosis experienced difficulties during embryo transfer, and a stiffer catheter core was used during the procedure. The pregnancy of this case was located in the right posterior myometrium, protruding toward the serosal layer. This was considered a result of embryo implantation directly into the uterine myometrium, leading to abnormal placentation.

The impact of BMI on pregnancy outcomes has increasingly drawn attention.^[27]^ Obesity is associated with multifaceted adverse effects on pregnancy outcomes. In the present study, the median BMI among the 8 patients was 25.5. To date, no reported studies have examined the association between obesity and IMP.

### 4.2. Clinical characteristics

The early clinical manifestations of IMP are atypical, often with no obvious symptoms. As the pregnancy progresses, it may lead to lower abdominal pain and uterine rupture. There have been case reports documenting uterine rupture occurring at 3 months’ gestation, 4 months’ gestation, and at 19 weeks’ gestation.^[28]^ To date, only 1 case has been reported in which uterine rupture at 30 weeks resulted in a live-born neonate.^[8]^ Statistical data indicate that uterine rupture during pregnancy predominantly occurs between gestational weeks 11 and 30. Research indicates that gestational age exceeding 10 weeks is an independent risk factor for hysterectomy.^[29]^

In the present study, none of the 8 patients had significant abdominal pain prior to diagnosis, with only 2 cases presenting minimal vaginal bleeding. None had experienced uterine rupture before treatment.

### 4.3. Diagnosis

IMP is a rare form of EP, with atypical early clinical manifestations, often leading to misdiagnosis as tubal pregnancy, cornual pregnancy, gestational trophoblastic tumors, or other diseases. Early diagnosis and treatment of IMP are critical to prevent uterine rupture and preserve fertility.

Diagnostic approaches rely on clinical presentation and imaging studies. Advances in ultrasound have significantly improved early detection, reducing the risk of life-threatening hemorrhage from ruptured gestational lesions or necessitating hysterectomy. Ultrasound may reveal heterogeneous masses or gestational sac echoes within the myometrium, though early misdiagnosis or localization inaccuracies can occur. For cases where ultrasound is inconclusive, MRI offers specific imaging features to clarify the relationship between the gestational sac and endometrium, aiding precise diagnosis and treatment planning. When ultrasound and MRI cannot confirm the diagnosis, surgical exploration (combined laparoscopy and hysteroscopy) is recommended for definitive diagnosis and intervention.^[9]^

In the present study’s 8 cases of embryo transfer-associated IMP, symptoms emerged between 23 and 54 days post-transfer. Preoperative ultrasound identified EP in 5 cases and IMP in 3 cases, with fetal cardiac activity observed in 3 cases. One case diagnosed at 54 days was considered to be a delayed diagnosis due to a large gestational mass. Early postconception ultrasound screening is emphasized to enable timely diagnosis and intervention.

Elevated serum β-HCG levels (ranging from 1271 to 106,910 mIU/mL) were observed, with most exceeding 6000 mIU/mL at admission.

### 4.4. Treatments

Current clinical approaches for IMP include surgical treatment, ultrasound-guided aspiration, and fetal reduction combined with local or systemic medication for embryo reduction. The choice of treatment must be based on a comprehensive analysis of the patient’s condition, including serum HCG levels, the size and location of the gestational lesion.

#### 4.4.1. Surgical treatments

Surgical treatment options include laparoscopic or hysteroscopic surgery for the removal of myometrial gestational lesions, or high-intensity focused ultrasound ablation as a noninvasive treatment.

The choice of surgical approach depends on the location of the gestational lesion: hysteroscopic surgery is preferred for lesions protruding into the uterine cavity, while laparoscopic surgery is indicated for lesions extending into the serosa. For cases of IMP with large lesions, thin surrounding myometrial walls, elevated serum HCG levels (high risk of rupture), or existing rupture, surgical intervention is mandatory.

If the IMP lesion is large but carries a low immediate rupture risk, and direct surgery poses a high risk of excessive intraoperative bleeding, medical treatment with MTX and/or mifepristone may be administered to reduce embryonic activity before subsequent surgical removal, thereby minimizing hemorrhage risk during surgery.

In cases of ruptured large IMP lesions with severe uterine wall destruction, significant intraoperative bleeding, and no fertility requirements, total hysterectomy is recommended. For patients desiring future fertility, lesion resection combined with uterine repair^[30]^ or high-intensity focused ultrasound ablation (a noninvasive treatment)^[31]^ may be considered.

Combined hysteroscopic and laparoscopic surgery allows comprehensive evaluation of the uterine cavity, prevents residual gestational tissue from penetrating the endometrium, and enables intraoperative administration of MTX under direct visualization to reduce embryonic activity prior to definitive removal of the lesion.^[32]^

For high-risk cases involving large lesions and significant bleeding risk, uterine artery embolization, such as cisplatin perfusion embolization of bilateral uterine arteries, can be employed to manage intramural myometrial pregnancies.^[33]^ Alternatively, temporary occlusion of uterine arteries during surgery, as used in other uterine procedures, may reduce intraoperative bleeding while minimizing impact on ovarian function.^[34]^

#### 4.4.2. Drug therapy

Drug therapy includes systemic and local administration. Systemic administration of MTX and Mifepristone is primarily indicated for cases with a confirmed diagnosis, lesion diameter <4 cm, serum HCG < 2000 IU/L, and no underlying liver/kidney disease,^[35]^ or for small intramural myometrial pregnancies detected only via ultrasound or other imaging studies but not identifiable during hysteroscopic/laparoscopic exploration.

*Local drug therapy*: Ultrasound-guided MTX injection: MTX is often injected directly into the gestational sac of IMP or its surrounding tissue under ultrasound guidance. During medication treatment, serum HCG levels should be regularly monitored.

Studies indicate that intra-sac administration of MTX achieves more ideal effects compared with intramuscular injection and has a lower incidence of MTX-related adverse effects compared with the intramuscular group. This suggests that ultrasound-guided intra-sac administration preserves endocrine and ovarian function better than conventional intramuscular injection and reduces drug-related risks.^[36]^

*Intrauterine intervention for IMP*: The lesion in the gestational sac of IMP is destroyed via hysteroscopic puncture under ultrasound guidance, combined with local intraoperative application and systemic postoperative administration of MTX, mifepristone, or traditional Chinese medicine (TCM).^[10]^

For large gestational lesions with high hemorrhage risk, direct intraoperative visualization and injection of MTX into the lesion to reduce embryonic activity is preferred before subsequent hysteroscopic or laparoscopic surgery.

*TCM therapy*: TCM demonstrates certain efficacy in treating ectopic pregnancies, particularly in early-stage tubal pregnancies under specific conditions.^[37]^

All 8 patients in the present study were confirmed via combined hysteroscopic and laparoscopic exploration. Cases with gestational lesions slightly protruding into the uterine cavity primarily underwent hysteroscopic resection of the gestational lesion, whereas lesions protruding into the uterine serosa were treated with laparoscopic resection. For cases where the lesion neither protruded into the uterine cavity nor into the serosa, ultrasound-guided local drug injection was the primary treatment approach.

In the early stages, diagnosis was delayed due to a lack of experience. For Case 1, the gestational lesion was relatively large at diagnosis, requiring a prolonged treatment duration. The patient initially underwent combined hysteroscopic and laparoscopic exploration with lesion injection, followed by a second surgical procedure to remove the lesion after abdominal pain occurred postdrug therapy.

For Case 2, ultrasound preoperatively diagnosed an interstitial pregnancy, but no gestational lesion was found during surgical exploration. This patient underwent ultrasound-guided reduction of the gestational lesion and local drug administration. A slow decline in serum HCG levels postoperatively was suspected to correlate with no lesion removal and low-dose MTX administration during the ultrasound-guided puncture.

For the remaining 6 cases, surgical treatment achieved rapid serum HCG decline, minimal intraoperative blood loss, and shorter time to normal HCG levels.

For IMP, early case reports documented life-threatening complications, such as uterine rupture, hemorrhagic shock, and hysterectomy, which led to the loss of reproductive function. These outcomes were primarily attributed to limited awareness of the disease, delayed diagnosis and treatment, and the use of less advanced ultrasound diagnostic techniques. With the advancement of medical science, such severe adverse outcomes have largely disappeared. In the present study, no cases of uterine rupture, hysterectomy, or hemorrhagic shock were observed. Eight patients were followed up for postoperative pregnancy outcomes in the present study: 1 case resulted in a spontaneous EP, 2 did not achieve subsequent pregnancy, and 5 became pregnant after FET following surgery. Among these 5, 3 were delivered at term via cesarean section, 1 was delivered vaginally, and 1 experienced an early missed abortion.

## 5. Limitations

First, the retrospective design and relatively small sample size (8 cases) limit the generalizability of the findings. These findings are purely descriptive observations and do not provide evidence of association or causation. IMP is a rare clinical entity; the small cohort restricts the ability to perform subgroup analyses or identify statistically significant risk factors and treatment outcomes. Second, the absence of a control group of patients undergoing embryo transfer without IMP limits the ability to identify potential predictive factors or causative associations, such as embryo quality, catheter type, or endometrial thickness. Third, all diagnoses were confirmed through combined hysteroscopic and laparoscopic exploration; some early cases lacked preoperative imaging precision, affecting diagnostic consistency. Long-term follow-up data on reproductive outcomes were incomplete in some patients, which may underestimate or overlook late complications or fertility-related consequences.

## 6. Future perspectives

ART continues to evolve and become more widely used; rare complications such as IMP, may become recognized. Future research could focus on large-scale, multicenter prospective studies to better characterize the incidence, risk factors, and optimal management of IMP following embryo transfer. In particular, investigating the role of embryo transfer techniques, including catheter type, depth of transfer, and uterine contractility, may help identify modifiable procedural risk factors. Further studies are needed to explore the potential impact of adenomyosis, previous uterine surgeries, and endometrial receptivity on the risk of abnormal implantation within the myometrium. Advances in high-resolution imaging modalities, such as 3-dimensional transvaginal ultrasound and MRI, may improve early and accurate diagnosis, reducing the risk of misdiagnosis or delayed treatment. The development of standardized diagnostic criteria and treatment algorithms, including the integration of conservative pharmacologic approaches with fertility-preserving surgical techniques, will guide individualized patient care. Long-term follow-up of patients treated for IMP is needed to assess reproductive outcomes and inform fertility counseling. Understanding the pathophysiology and clinical course of IMP will enhance early detection, minimize complications, and improve maternal and reproductive health outcomes. Our previous study supports the present study in favor of clinical analysis of intramural pregnancy after embryo transfer.^[38]^

## 7. Conclusion

IMP is a high-risk EP with an atypical clinical presentation. Risk factors include adenomyosis, a history of tubal EP surgery, a history of intrauterine adhesion surgery, and embryo transfer methods. Early diagnosis and timely treatment prevent uterine rupture and preserve fertility. Treatment selection is guided by lesion location, size, and serum HCG levels, with a comprehensive approach that prioritizes surgical lesion removal and is supplemented by pharmacological therapy.

## Acknowledgments

The authors would like to acknowledge the technical support provided by The Reproductive Hospital of Guangxi Zhuang Autonomous Region.

## Author contributions

**Conceptualization:** Juan Wang.

**Methodology:** Xiaohong Li.

**Data curation:** Fengxia Liu.

**Formal analysis:** Yourong Cao.

**Investigation:** Bin Zhang.

**Supervision:** Yi Mo.

**Writing – original draft:** Arshad Mehmood.

**Writing – review & editing:** Arshad Mehmood.

## Correction

The term “interstitial” has been replaced with “intramural” in some parts of the text.
